# Serotonergic drug repurposing in multiple sclerosis: A new possibility for disease-modifying therapy

**DOI:** 10.3389/fneur.2022.920408

**Published:** 2022-07-22

**Authors:** Mikhail Melnikov, Dmitriy Kasatkin, Anna Lopatina, Nikolay Spirin, Alexey Boyko, Mikhail Pashenkov

**Affiliations:** ^1^Department of Neuroimmunology, Federal Center of Brain Research and Neurotechnology of the Federal Medical-Biological Agency of Russia, Moscow, Russia; ^2^Department of Neurology, Neurosurgery and Medical Genetics, Pirogov Russian National Research Medical University, Moscow, Russia; ^3^Laboratory of Clinical Immunology, National Research Center Institute of Immunology of the Federal Medical-Biological Agency of Russia, Moscow, Russia; ^4^Department of Neurology, Neurosurgery and Medical Genetics, Yaroslavl State Medical University, Yaroslavl, Russia

**Keywords:** fluoxetine, Th17-cells, neuroimmune interaction, drug repurposing, multiple sclerosis

## Abstract

Investigation of neuroimmune interactions is one of the most developing areas in the study of multiple sclerosis pathogenesis. Recent evidence suggests the possibility of modulating neuroinflammation by targeting biogenic amine receptors. It has been shown that selective serotonin reuptake inhibitor fluoxetine modulates innate and adaptive immune system cells' function and can reduce experimental autoimmune encephalomyelitis and multiple sclerosis severity. This brief report discusses the immune mechanisms underlying the multiple sclerosis pathogenesis and the influence of fluoxetine on them. The retrospective data on the impact of fluoxetine treatment on the course of multiple sclerosis are also presented. The results of this and other studies suggest that fluoxetine could be considered an additional therapy to the standard first-line disease-modifying treatment for relapsing–remitting multiple sclerosis.

## Introduction

The neuroimmune interactions are among the most developing areas in the study of multiple sclerosis (MS) pathogenesis (1). Serotonin [5-hydroxytryptamine (5-HT)] is a direct mediator of this interaction.

On the one hand, the influence of 5-HT on MS pathogenesis could be mediated by its involvement in the formation of neuropsychological symptoms of MS (depression, cognitive impairments, and fatigue). On the other hand, 5-HT may regulate the ≪gut-brain≫ axis and modulate immune cell activity and cytokine production (2, 3). It is known that immune cells of both the innate and adaptive immune systems express serotonergic receptors (3). Furthermore, according to some studies, several immune cells may produce 5-HT, which suggests serotonergic autoregulation of immune cell function (4).

In this regard, much attention is drawn to the potential ability to inhibit neuroinflammation by treatment with serotonergic drugs. *In vitro* and *in vivo* studies have shown that selective serotonin reuptake inhibitors (SSRIs) can reduce EAE and MS severity (5). However, the immune mechanism of the anti-inflammatory effect of SSRIs on MS pathogenesis needs further study. Recent evidence suggests that the SSRI fluoxetine suppresses interleukin-17 (IL-17) and interferon-γ (IFN-γ) production by T-cells *via* 5-HT_2B_-receptor activation in patients with relapsing–remitting MS (6). Also, activation of 5-HT_2B_ receptors on dendritic cells has been reported to inhibit dendritic cell-mediated activation of Th17- and Th1-cells in healthy subjects (7).

This brief report discusses the immune mechanisms underlying MS pathogenesis and the impact of fluoxetine on them, with a focus on the function of Th17-cells as crucial players in CNS autoimmunity. In addition, retrospective data on the influence of fluoxetine treatment on MS course are also presented, which confirm the potential clinical efficacy of fluoxetine as an additional therapy to standard first-line disease-modifying treatment (DMT) for relapsing–remitting MS.

## The immune mechanisms underlying neuroinflammation and axonal degeneration in MS

The main hypothesis of MS is a violation of immune tolerance and active penetration of the myelin-sensibilization immune cells into the central nervous system (CNS) (≪outside-in≫ theory) (8). For a long time MS has been considered a predominantly T-cell mediated disease. Among the T-cells, the most attention is drawn to the CD4^+^ T-cells subsets, in particular, Th17- and Th1-cells, which produce pro-inflammatory cytokines IL-17 and IFN-γ, respectively (9). The pathogenetic role of Th17-cells in MS could be explained by their ability to migrate into the CNS through the blood-brain barrier by the expression of chemokine receptor-6 [CCR6 (CD196)] and IL-17 production (10). The involvement of Th17-cells in the pathogenesis of other inflammatory diseases of the CNS (Parkinson's disease, depression) has been shown (11). According to literature data, Th17-cells are among the most critical targets for the DMT of MS (12).

However, the data of past decades suggest that B-cells also play a significant role in MS pathogenesis (13). It is known that B-cells are not only able to differentiate into plasma cells and produce antibodies, but also produce cytokines and present antigens (13). The high diagnostic value of oligoclonal IgG bands and free immunoglobulin κ- and λ-light chains in the cerebrospinal fluid, as well as the clinical efficacy of anti-B-cell therapy in patients with MS confirm the involvement of B-cells in MS pathogenesis (13). Although the mechanism of B-cells migration in the CNS is not sufficiently investigated, it has been demonstrated that B-cells are directed into the CNS by chemokine signals. The primary role in the migration of B-cells may belong to CXCL13. However, the blockade of adhesion molecules, such as VLA-4, ICAM-1, and ALCAM can also prevent B-cells migration through brain-derived endothelial cells *ex vivo* (14).

The cells of the innate immune system also migrate into the CNS. In particular, it has been shown that monocytes can penetrate the blood–brain barrier, differentiate into macrophages, and mediate autoimmune inflammation in the brain. Furthermore, among the immune cells, present in chronic and acute EAE and MS lesions, macrophages are the most common (15). It has been shown that the migration of monocytes into the CNS corresponds to the clinical progression of the EAE. In contrast, the blockade of CCL2 and CCR2 receptors on monocytes reduces EAE symptoms (16). A few studies have shown that depletion of circulating blood monocytes attenuates EAE development and reduces its severity. In addition, the therapeutic effect of monocyte depletion in EAE has been shown (17).

At the same time, there are resident immune cells in the CNS. In particular, in the CNS, resident macrophages form microglia, which are capable of presenting antigens and producing cytokines and, depending on their phenotype, may participate in the development of autoimmune inflammation or maintenance of immunological tolerance (15).

A critical pathogenetic role in EAE and MS can be played by peripheral monocytes and microglial cells. Activation of microglial cells has been shown to prevent the development of clinical symptoms and migration of monocytes into the CNS from the periphery in EAE. At the same time, at the peak of the disease, microglial cells make up no more than 37% of the total number of macrophages and CD11c^+^-dendritic cells found in foci of demyelination, which may indicate the participation of microglia and macrophages at different stages of the pathological process. It appears that the activation of microglial cells likely occurs at an early stage of neuroinflammation (18). Other studies confirm these data. Thus, histological examination of the brain tissue of patients with MS revealed the activation of microglial cells in the normal-appearing white matter (before cellular infiltration from the periphery and damage to myelin), which also indicates early activation of microglia (15).

It is important to note that in EAE and MS, along with demyelination, macrophages mediate axonal degeneration, which underlies the progressive forms of MS (primary- and secondary-progressive MS) and causes the development of neurological disability (19). In several studies, the correlation between the accumulation of the β-amyloid precursor protein (a marker of axonal damage) in damaged axons and the number of macrophages at the site of injury in progressive MS was shown (20–22).

The role of B-cell in the development of neurodegeneration and MS progression is also discussed. It was shown that ectopic lymphoid follicles, represented mainly by B-cells, are formed in the meninges of patients with secondary-progressive MS. It is supposed that such B-cell structures are particularly likely to contribute to cortical demyelination and disease progression. Moreover, recent findings suggest that meningeal B-cell follicles are formed at the earliest stages of MS, which may explain neurodegenerative changes at the disease onset (23, 24). It is important to note that among more than fifteen disease-modifying drugs of MS, only ocrelizumab (the monoclonal antibody to CD20) has clinical efficacy in primary-progressive MS, suggesting the role of B-cells in MS progression (25).

In general, data suggests the critical role of the cells of both the innate and adaptive immune systems in demyelination and neurodegeneration in MS. It is also important to consider the different roles of the peripheral immune cells and resident immune cells of the CNS. Apparently, their involvement occurs at the different stages of the disease: initiation of autoimmune inflammation, which can be mediated by the microglia activation, and the effector phase of the disease mediated by the immune cells infiltrating into the CNS through the blood-brain barrier from the periphery.

## The influence of fluoxetine on EAE and MS pathogenesis

The investigation of the immunomodulatory effect of biogenic amines is one of the most developing areas in MS pathogenesis study. Therapeutics targeting the biogenic amines receptor can modulate the functioning of the immune system. Recent evidence suggests that treatment with selective serotonin reuptake inhibitors (SSRIs) may improve the course of EAE and MS (3). The immune mechanisms underlying this effect continue to be studied. Considering the crucial role of Th17-immune response in MS and other inflammatory diseases of the CNS, the influence of SSRIs on Th17-cells function has drawn much attention. However, the effect of SSRIs on Th17-cells needs to be clarified. There are only some data on the impact of SSRIs on Th17-immune response.

In particular, Bhat et al. (26) showed that *in vivo* and *in vitro* treatment with fluoxetine reduces IL-17 and IFN-γ production by stimulated splenocytes and CD3^+^ T-cells. Sales et al. (27) reported the inhibitory effect of SSRI fluoxetine on Th17-cells in MS. Thus, a 6-month treatment with fluoxetine (20 mg/day) attenuated the secretion of IL-17 by stimulated CD4^+^ and CD8^+^ T-cells in relapsing–remitting MS patients with depression (27).

In line with these data, our recent study has shown that fluoxetine (at 10^−6^ M) suppresses pro-inflammatory Th17-immune response in MS *in vitro*. Fluoxetine has been found to reduce IL-17, IFN-γ, granulocyte-macrophage colony-stimulating factor (GM-CSF), and IL-21 production by CD4^+^ T-cells stimulated with anti-CD3/CD28-microbeads in patients with relapsing–remitting MS and healthy subjects without affecting immune cell viability and proliferative response. It has also been found that the 5-HT_2B_-receptor activation can mediate this effect in patients with MS. In addition, the direct inhibitory effect of a 5-HT_2B_-receptor agonist (BW723C86) on IL-17, IFN-γ, and GM-CSF by activated T-cells in MS and healthy subjects was demonstrated. It is important to note that 5-HT also suppressed cytokine production by CD4^+^ T-cells in both groups. However, this effect of 5-HT was achieved at a concentration of 10^−4^ M (a hundred times more than the concentration of fluoxetine). Herewith, there was no effect of 5-HT_2B_-receptor antagonist (RS127445) on the inhibitory effect of 5-HT on cytokine production in both groups. Finally, fluoxetine did not increase 5-HT production by stimulated CD4^+^ T-cells (6). These data suggest that the immune effect of fluoxetine may be independent of inhibition of 5-HT reuptake.

Another study showed the inhibitory effect of fluoxetine (at 10^−6^ M) on IL-6 and IL-1β production by lipopolysaccharide-activated dendritic cells in patients with relapsing–remitting MS and healthy subjects. Again, 5-HT_2B_-receptor antagonist (RS127445) reduced fluoxetine-mediated IL-1β suppression in both groups and IL-6 in healthy subjects, while 5-HT_2B_-receptor agonist (BW723C86) enhanced the inhibitory effect of fluoxetine on IL-6 in both groups (28).

The anti-inflammatory effect of the 5-HT_2B_-receptor agonist (BW723C86) on dendritic cells-mediated Th17-immune response in humans was reported by Szabo et al. (7), who showed that the activation of 5-HT_2B_-receptor on CD1a^+^-dendritic cells with a specific agonist (BW723C86) reduces their ability to activate autologous naive Th17- and Th1-cells. In addition, 5-HT_2B_-receptor activation suppressed IL-6 and IL-12 production by activated dendritic cells. At the same time, anti-5-HT_2B_-receptor monoclonal antibody blocked this effect (7).

Thus, the influence of fluoxetine on Th17-immune response can be mediated by the direct impact on Th17-cells as well as through suppression of antigen-presenting cells. It can be assumed that the immunomodulatory effect of fluoxetine on Th17-cells is associated with the activation of the 5-HT_2B_-receptor.

Fluoxetine has also been shown to have an anti-inflammatory effect on both peripheral macrophages and central microglia (3). In a recent review, Mariani et al. (29) summarized the impact of different antidepressants on microglia activation. Their data suggest that SSRIs prevent microglial activation, including reduction of microglial reactivity and reduction of immune and oxidative stress products, and they inhibit macrophages/microglial M1-polarization (29, 30). The inhibitory effect of fluoxetine *via* 5-HT_2B_-receptor on the activation of A1-reactive astrocytes has also been shown (31).

In general, there is more and more data confirming the anti-inflammatory effect of fluoxetine in MS. It is important that fluoxetine may provide this effect in the periphery and directly in the CNS.

## The influence of fluoxetine on relapsing–remitting MS course

To evaluate the influence of fluoxetine on MS course, we conducted a pilot retrospective non-interventional, non-comparator study aimed at evaluating the possibility of using SSRI fluoxetine in patients with relapsing–remitting MS with the suboptimal response to the first-line DMT (IFN-β or glatiramer acetate).

Seventy patients (56 females) with a documented diagnosis of MS according to McDonald criteria were examined (32). All patients had a relapsing–remitting form of the disease. The average age was 35.2 years and the duration of the disease was 4.2 years. All patients were subjected to a standard neurological examination with EDSS score (the median EDSS score – 4) (33). All patients were examined during clinical remission without MRI activity. All patients had been treated with glatiramer acetate (*n* = 20) or IFN-β (IFN-β1a, *n* = 20; IFN-β1b, *n* = 30) for more than 1 year. All patients are characterized by the suboptimal response to the therapy determined by Modified Rio Score (data not shown) (34). All patients had no cognitive impairments, according to Montreal Cognitive Assessment, and had mild or moderate depression according to the Beck Depression Inventory (≥19 points) (35, 36). The study design is presented in Table 1. In all clinical cases, the last 2 years of therapy were analyzed (1 year under the treatment with DMT and 1 year with DMT and fluoxetine).

**Table 1:** The number of clinical visits and evaluated characteristics.

**Visit, No**.	**Evaluated characteristics**				
	**Relapses**	**EDSS**	**MRI activity**	**Depression**	**Cognitive impairments**
Visit 1 (baseline, starting therapy with fluoxetine)	+	+	+	+	+
Visit 2 (1 year of treatment with DMT and fluoxetine)	+	+	+	+	+

The statistical analysis of the results was performed using Prizm 6 software. The Wilcoxon signed-rank test was used to compare the two groups. Differences were considered statistically significant at *p* < 0.05.

All patients/participants signed the written informed consent to participate in this study. The study was approved by the Ethics Committee of the Pirogov Russian National Research Medical University (Protocol No. 209).

**Figure 1:**
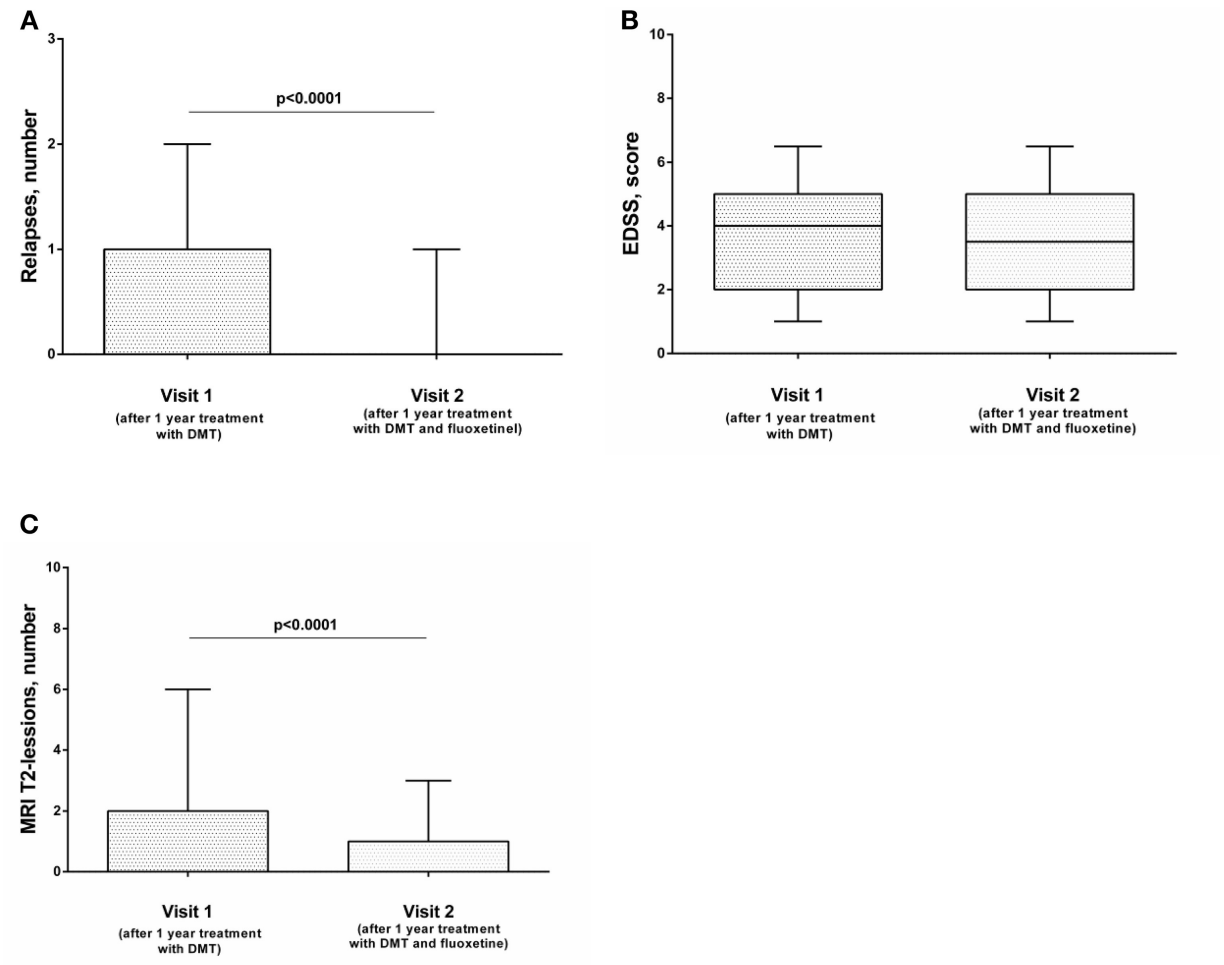
The influence of combined therapy (DMT and fluoxetine) on the frequency of exacerbations **(A)**, EDSS score **(B)**, and MRI activity **(C)** of the disease in patients with relapsing–remitting MS (*n* = 70). The Wilcoxon signed-rank test was used to compare two groups. Horizontal lines on the graphs correspond to the median and whiskers indicate to the min. and max values. The median values were compared and the *p*-values are indicated in the figure.

After 1 year of combined therapy (DMT and fluoxetine), relapse rate decreased significantly (Figure 1A), while the EDSS level was comparable with baseline (Figure 1B). MRI activity also decreased significantly: numbers of new or newly enlarging T2 lesions were decreased significantly compared with the previous year (Figure 1C). The level of depression also decreased, but this effect was not significant (data not shown). The same effect was observed when we analyzed the data depending on the type of the therapy (Supplementary Figure 1), except data on the influence of therapy with IFN-β1a and fluoxetine on the frequency of relapses. Although the number of relapses was lower than after 1 year of monotherapy with IFN-β1a, the data were not statistically significant (Supplementary Figure 1B). However, this could be explained by the small sample size (*n* = 20).

The presented data are preliminary and have some limitations (small sample size, short duration of observation, and absence of the control group). In addition, there is no data on the effect of treatment with fluoxetine on immune response in patients with MS. Nevertheless, the results of our study correspond to the data from other studies *in vivo* and *in vitro*, confirming the anti-inflammatory effect of fluoxetine in autoimmune diseases (3, 5). In particular, similar effects of treatment with SSRIs was observed in rheumatoid arthritis and psoriasis (3). The therapeutic potential of SSRIs as a treatment of MS was previously studied during a clinical trial (37, 38). However, there was no convincing evidence of the clinical efficacy of fluoxetine in MS treatment. It is important to note that these studies focused on the monotherapy with fluoxetine for secondary-progressive MS, which is primarily associated with neurodegeneration, while fluoxetine may supposedly influence the neuroinflammatory component of the disease. In addition, patients who participated in these studies had not been treated with DMT for more than 6 months. Limitations of these studies, as well as the possible reasons for the absence of the clinical efficacy of fluoxetine were discussed by Grech et al. (39) and Mostert and De Keyser (40).

In line with Mostert and De Keyser, we suggest that fluoxetine may be more effective in relapsing–remitting MS in which autoimmune inflammation can prevail over neurodegeneration (40). In addition, we suppose that fluoxetine can be considered as an additional therapy to standard first-line DMT in patients with relapsing–remitting MS with suboptimal response. It can be suggested that fluoxetine can increase the efficacy of glatiramer acetate or IFN-β without switching to the second-line treatment. However, a randomized clinical trial with a control group is necessary.

## Conclusion and prospects

Despite the progress in DMT, MS treatment is still one of the main problems in clinical neurology. Modern, highly effective targeted therapy can reduce disease activity. However, the potent immunosuppressive impact of such therapy causes serious side effects. It is important to note that not all clinical cases with suboptimal responses to treatment with first-line DMT fully correspond to the criteria for switching to second-line DMT. In particular, the primary scale for efficacy assessment of first-line injectable therapy in MS is Modified Rio Score (Supplementary Table 1) (34). According to this scale, there are optimal and suboptimal responses. However, if we look more closely, we can find a small gap between these two points. For example, suppose that the patient has one to four T2-lesions on MRI or one mild relapse without MRI activity during the treatment with IFN-β or glatiramer acetate for more than 6 months, then in that case, he/she is not an optimal responder. At the same time, there are no criteria for switching to the second-line DMT. In this regard, the suboptimal response to first-line DMT is an important challenge for routine clinical practice. Therefore, the search for additional therapeutics that may enhance first-line drugs' efficacy is an important task.

Fluoxetine is one of the most often prescribed therapeutic agents for treating depression in MS. It is well known that depression is widespread in patients with MS and may aggravate MS courses. Furthermore, the same pathogenetic mechanisms may underlie these diseases and form one of the vicious circles of MS. In this regard, fluoxetine may affect MS pathogenesis by remitting depression and reducing stress-related exacerbations (41). At the same time, the direct anti-inflammatory effect of fluoxetine on Th17-immune response is also possible.

In a recent systematic review, Stamoula et al. (5) analyzed the available *in vitro* and *in vivo* data on the impact of antidepressants on EAE and MS pathogenesis and confirmed the potential anti-inflammatory effect of antidepressants. Therefore, if clinical trials demonstrate the effectiveness of SSRIs as an additional therapy for MS, the inclusion of such therapy in the treatment standards of MS should be considered.

## Data availability statement

The raw data supporting the conclusions of this article will be made available by the authors, without undue reservation.

## Ethics statement

The study was approved by the Ethics Committee of the Pirogov Russian National Research Medical University (Protocol No. 209). The patients/participants provided their written informed consent to participate in this study.

## Author contributions

MM: conceptualization, investigation, methodology, and writing—original draft preparation. DK and AL: investigation and writing—original draft preparation. NS: conceptualization. MP: writing—review and editing. AB: conceptualization and methodology. All authors agree to be accountable for the content of the work.

## Funding

This work was financially supported by the Russian Foundation for Basic Research (RFBR) and Moscow City Government according to the Project No. 21-315-70014. The funders had no role in study design, data collection and analysis, decision to publish, or preparation of the manuscript.

## Conflict of interest

The authors declare that the research was conducted in the absence of any commercial or financial relationships that could be construed as a potential conflict of interest.

## Publisher's note

All claims expressed in this article are solely those of the authors and do not necessarily represent those of their affiliated organizations, or those of the publisher, the editors and the reviewers. Any product that may be evaluated in this article, or claim that may be made by its manufacturer, is not guaranteed or endorsed by the publisher.
