# Supplementary material for: Serotonergic drug repurposing in multiple sclerosis: A new possibility for disease-modifying therapy
Source: Front Neurol. 2022 Jul 22;13:920408. doi: 10.3389/fneur.2022.920408 (PMC9355384; doi:10.3389/fneur.2022.920408)
Supplement: Supplementary file 1 [file Presentation_1.PPT]

## Slide 1
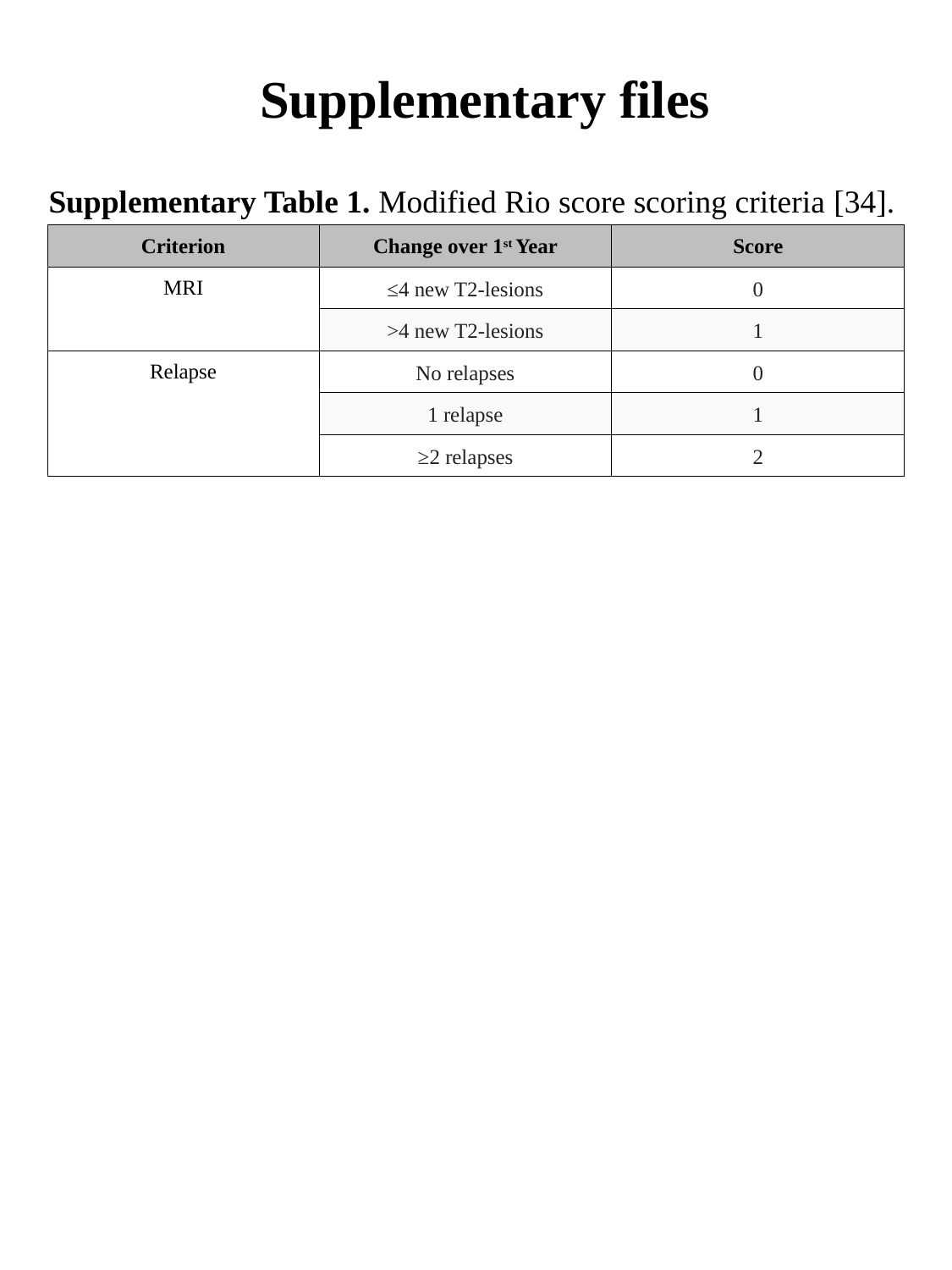

# Supplementary files
Supplementary Table 1. Modified Rio score scoring criteria [34]. [33].
| Criterion | Change over 1st Year | Score |
| --- | --- | --- |
| MRI | ≤4 new T2-lesions | 0 |
| | >4 new T2-lesions | 1 |
| Relapse | No relapses | 0 |
| | 1 relapse | 1 |
| | ≥2 relapses | 2 |

## Slide 2
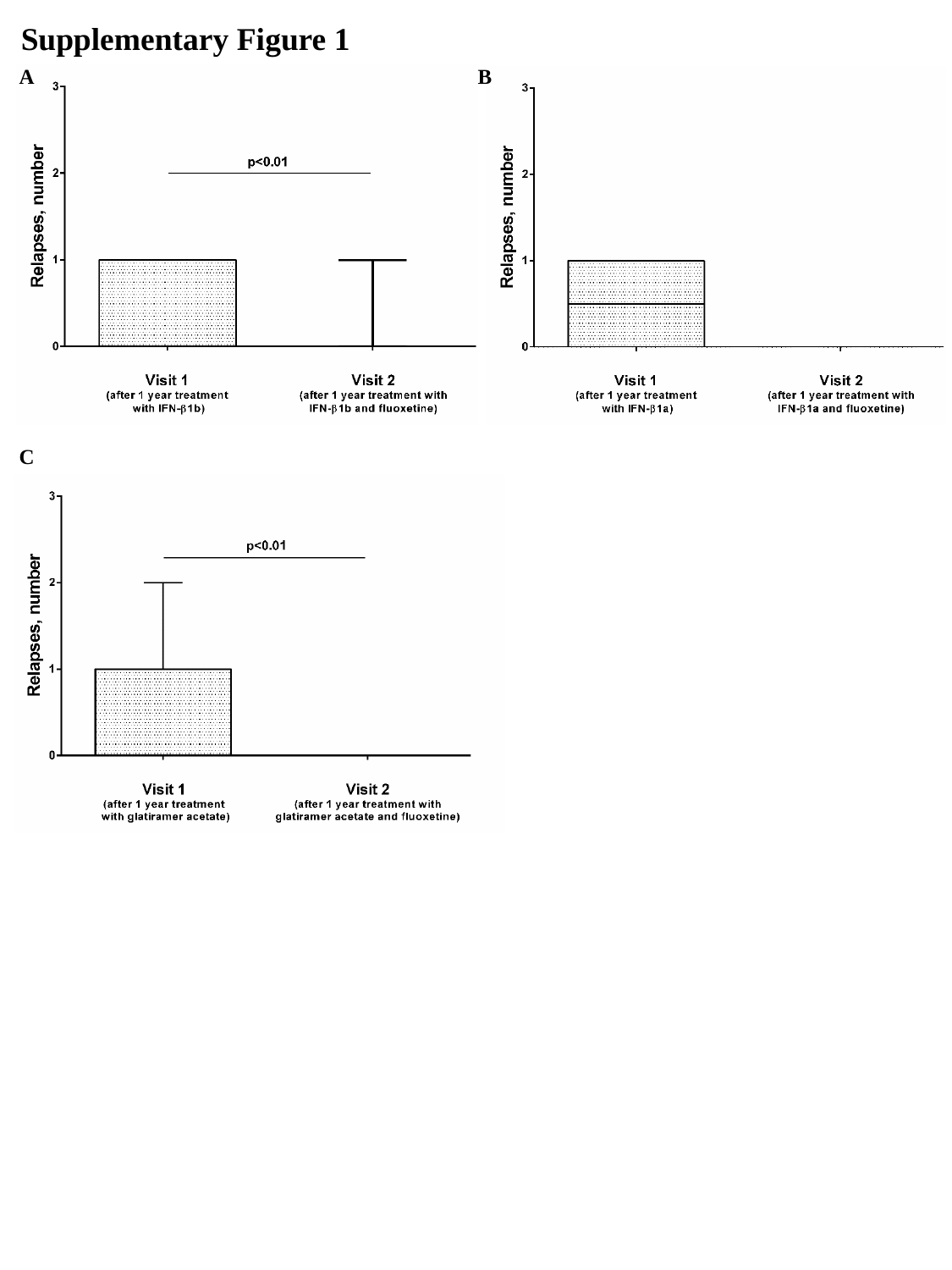

Supplementary Figure 1
A
B
C

## Slide 3
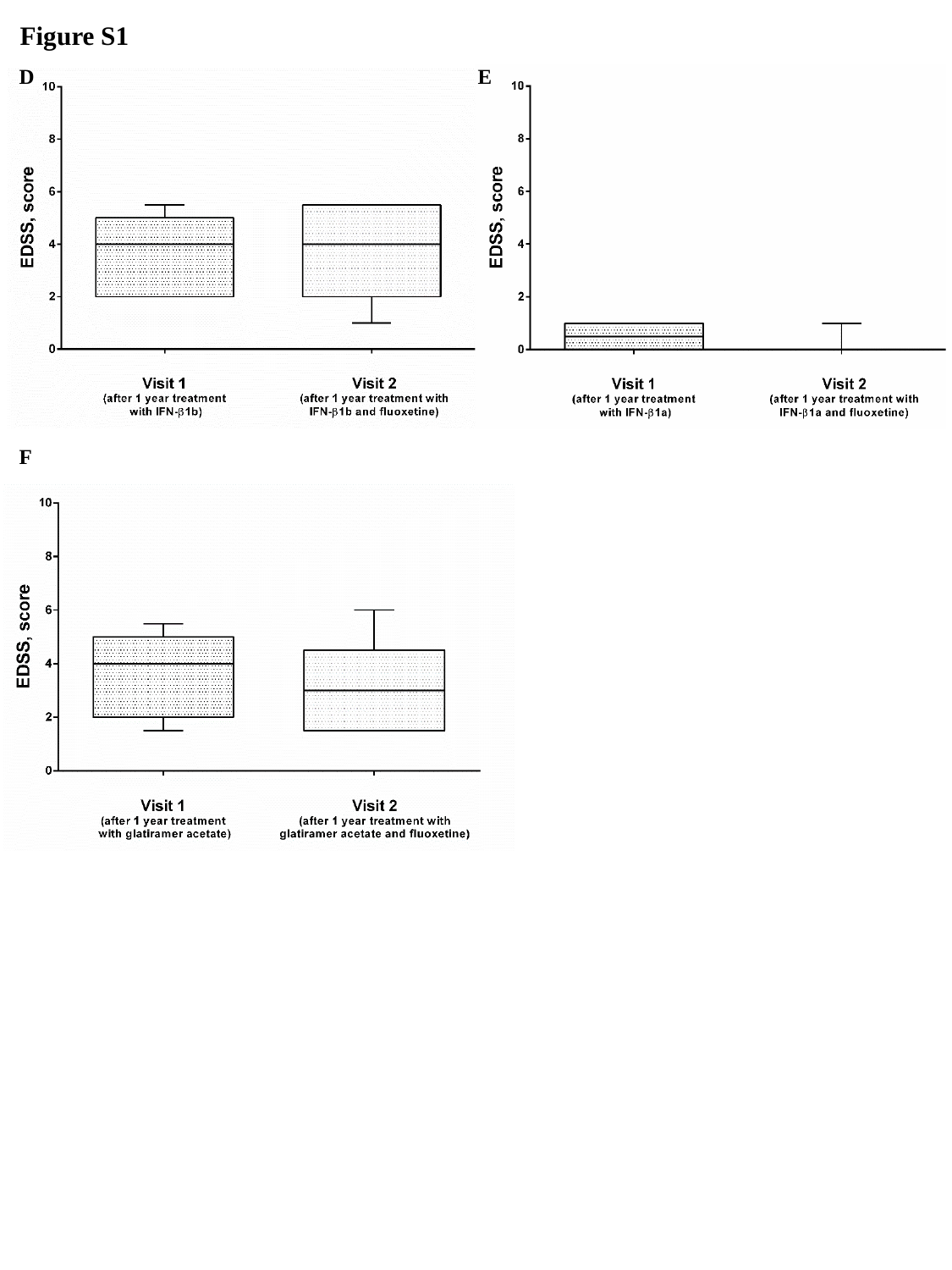

Figure S1
D
E
F

## Slide 4
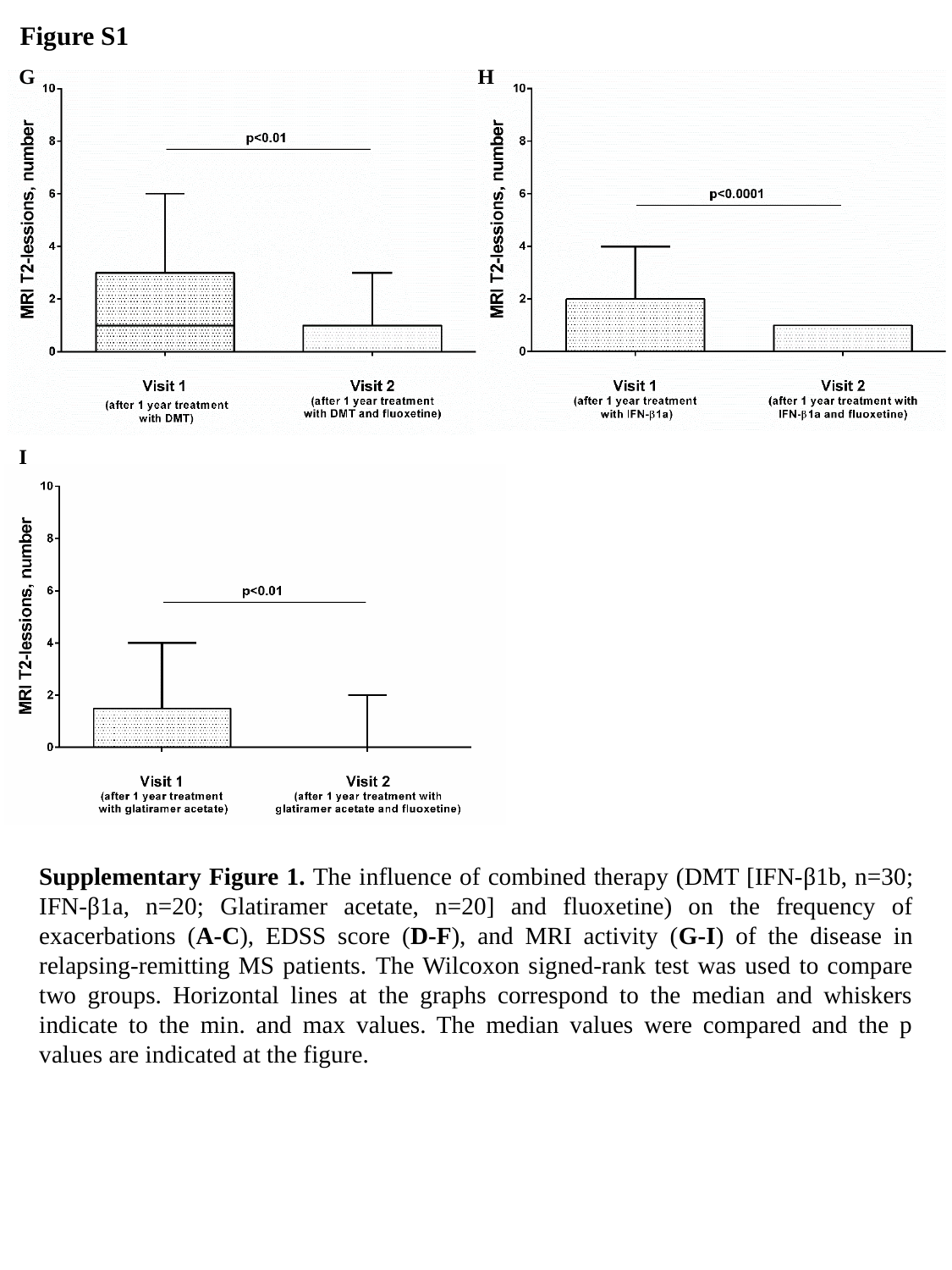

Figure S1
G
H
I
Supplementary Figure 1. The influence of combined therapy (DMT [IFN-β1b, n=30; IFN-β1a, n=20; Glatiramer acetate, n=20] and fluoxetine) on the frequency of exacerbations (A-C), EDSS score (D-F), and MRI activity (G-I) of the disease in relapsing-remitting MS patients. The Wilcoxon signed-rank test was used to compare two groups. Horizontal lines at the graphs correspond to the median and whiskers indicate to the min. and max values. The median values were compared and the p values are indicated at the figure.
